# An Investigation into the Re-Emergence of Disease Following Cessation of Antibiotic Treatment in Balb/c Mice Infected with Inhalational *Burkholderia pseudomallei*

**DOI:** 10.3390/antibiotics11101442

**Published:** 2022-10-20

**Authors:** Thomas R. Laws, Kay B. Barnes, Dominic C. Jenner, Alejandro Núñez, Mark I. Richards, Joanne E. Thwaite, Andreas Vente, David Rushton, Michelle Nelson, Sarah V. Harding

**Affiliations:** 1 Defence Science and Technology Laboratory, Porton Down, Salisbury SP4 0JQ, UK; 2 Animal and Plant Health Agency, Weybridge KT15 3NB, Surrey, UK; 3 MerLion Pharmaceuticals, 13125 Berlin, Germany; 4 Department of Respiratory Sciences, University of Leicester, Leicester LE1 7RH, UK

**Keywords:** *Burkholderia pseudomallei*, antibiotic, finafloxacin, co-trimoxazole, relapse, eosin

## Abstract

*Burkholderia pseudomallei* is the causative agent of melioidosis, a multifaceted disease. A proportion of the mortality and morbidity reported as a result of infection with this organism may be due to the premature cessation of antibiotic therapy typically lasting for several months. The progression of re-emergent disease was characterised in Balb/c mice following cessation of a 14 day treatment course of co-trimoxazole or finafloxacin, delivered at a human equivalent dose. Mice were culled weekly and the infection characterised in terms of bacterial load in tissues, weight loss, clinical signs of infection, cytokine levels and immunological cell counts. Following cessation of treatment, the infection re-established in some animals. Finafloxacin prevented the re-establishment of the infection for longer than co-trimoxazole, and it is apparent based on the protection offered, the development of clinical signs of disease, bodyweight loss and bacterial load, that finafloxacin was more effective at controlling infection when compared to co-trimoxazole.

## 1. Introduction

*Burkholderia pseudomallei,* the causative agent of the disease melioidosis, is endemic in tropical and sub-tropical parts of the world, where it is estimated to be the cause of 89,000 deaths per year [1]. Although it is an environmental organism, it can cause severe infections in humans following exposure and is classified by the Centres for Disease Control and Prevention (CDC) as a Category B biological agent. This is due to its high infectivity and natural resistance to many antibiotics [2].

Human infection can become established following exposure by a number of routes including inhalational, subcutaneous and ingestion [3]. The severity of the disease observed is dependent on many risk factors including the immune status of the individual, the infecting dose and the route of exposure, bacteria delivered as an aerosol establishing more severe infections, which are more likely to lead to pneumonia and septicaemia [4]. Melioidosis has a mortality rate of 35% and 26% in Thailand and Australia (two countries that routinely report cases), therefore effective diagnosis and early administration of antibiotics is very important [5,6]. Delayed or ineffective choices of treatment can result in severe disease that can be fatal [2,7].

Treatment of melioidosis is comprised of two phases, the acute or intensive phase which is typically 14 days of intravenously delivered ceftazidime or meropenem following by an ‘eradication’ phase which is months of orally delivered therapy, typically 3–6 months of co-trimoxazole [8]. In some cases of melioidosis, co-trimoxazole is also used as a therapeutic adjunct, during the acute phase, in infections considered more difficult to treat, including osteomyelitis and infections of the central nervous system [9,10]. In most cases, this successfully treats the patient, however this is dependent on the compliance of the individual regarding completion of the antibiotic course in the face of the potential for adverse side effects.

A recent retrospective study of 234 cases of melioidosis spanning 5 years in Australia identified 4.7% of patients that relapsed with disease or were reinfected with another *B. pseudomallei* strain [11]. These cases were in patients that had infections that were considered more difficult to treat including osteomyelitis, septic arthritis and deep-seated abscess. A similar study in Thailand reported a relapse rate of 9.7% from 889 cases [12]. In this study, the main contributing factor for relapse was the length and choice of oral antibiotic therapy for the eradication phase.

The pathogenesis of *B. pseudomallei* strain K96243 delivered as an aerosol to Balb/c mice has been characterised by many different groups. The LD_50_ of this strain has been calculated as approximately 4 CFU and can result in mice succumbing to disease by 72 h post-challenge, due to rapid dissemination of bacteria throughout the body and high levels of bacteria in key organs [13,14,15]. Although the usefulness of this sensitive mouse model has been questioned, acute disease in humans accounts for as high as 85% of cases with a similar clinical presentation [4,16].

The efficacy of co-trimoxazole against *B. pseudomallei* infection in Balb/c mice has also been evaluated extensively. One study demonstrated that when delivered at 24 h post-challenge, all mice were protected until day 21 post-challenge (when the experiment was terminated) and the spleens, livers and lungs were clear from colonising bacteria [17]. Similar results were found in another study when treatment was initiated at 6 h post-challenge. Complete protection was offered at day 21 post-challenge; however, when animals were treated with a course of dexamethasone from day 39 post-challenge, they succumbed to infection and *B. pseudomallei* was isolated from all survivors [18]. These data suggest that, although co-trimoxazole is efficacious and offers protection against acute disease, it is unable to effectively kill a proportion of bacteria that are able to survive the treatment phase. This may be due to the antibiotic being unable to kill the type of bacterial cells that may be formed in the course of an infection (e.g., persister cells), its poor penetration into tissues where the bacteria are residing or its limited antibiotic activity due to the environmental conditions to which it is exposed.

*B. pseudomallei* can survive in many cells and locations within a host, and it is difficult to treat in such environments that can be naturally acidic, or can be altered following an insult. For example, the pH of airway surface liquid and the alveolar subphase fluid of the lung can be reduced following infection [19]. In addition, inflammation (that can occur as a result of an infection), can cause metabolic acidosis which also results in a lowered pH [20,21]. Treating infections in these acidic environments can be less effective than at neutral pH, particularly with antibiotics from the fluoroquinolone class and the folic acid synthesis inhibitors [22,23,24]. In addition, pH has been shown to modulate the adherence and invasion of *B. pseudomallei* in an A549 tissue culture model. Optimal adherence and invasion for strain K96243 was pH 6.7 and pH 6.3 [25].

Finafloxacin is a fifth-generation fluoroquinolone that has in vitro activity against a range of Gram negative and Gram-positive organisms, including those of public health concern, and those of biodefence interest [26,27,28,29]. This in vitro activity has been shown to be superior in infection-relevant acidic conditions and similar to comparator antibiotics in neutral conditions, possibly due to the rapid accumulation of the antibiotic within cells and the slow efflux rate [24,30]. Efficacy has also been demonstrated with finafloxacin against inhalational infections with *B. pseudomallei*, *Francisella tularensis, Yersinia pestis* and *Coxiella burnetii* in vivo and it is being considered as a potential candidate for the treatment of melioidosis [31,32,33,34].

Previous work at Dstl has shown that following an inhalational challenge with *B. pseudomallei*, at day 15 post-challenge (1 day following cessation of finafloxacin initiated at 24 h post-challenge and delivered for 14 days), the spleens, livers and lungs harvested from mice were clear from colonising bacteria (i.e., no bacterial cells could be cultured on solid agar or in liquid media) [31]. Other groups of animals were treated and observed for a further 14 days. Some animals treated with finafloxacin started to relapse and succumbed to disease. Again, this suggests that a proportion of the bacterial cells are able to survive in a different niche in the host or form a cell type that cannot be detected using routine culturing methods. Understanding more about what is happening within the host from when treatment is stopped and disease re-emerges may help design better treatment strategies.

This work details an investigation into the re-emergence of disease following cessation of antibiotic treatment in Balb/c mice infected with inhalational *B. pseudomallei*. Mice were treated with 14 days of orally delivered finafloxacin or co-trimoxazole, 24 h post-challenge with *B. pseudomallei*. Following cessation of treatment, groups of animals were culled weekly for seven weeks, their organs harvested and the homogenates cultured for bacterial enumeration or analysed by immunoassay. This study aimed to characterise the progression of re-emergent disease in antibiotic treated animals and determine the site of bacterial re-emergence post-treatment cessation.

## 2. Results

### 2.1. Premature Cessation of Antibiotics Results in Resurgence of Disease

Mice were infected with *B. pseudomallei* strain K96243 via the inhalational route with a mean retained dose of 142 CFU (range 78–197 CFU). The challenge was lethal, all control animals (treated with vehicle) succumbed to infection by day 4 post-challenge. Antibiotic treatment was initiated at 24 h post-challenge. At this point in time, mice displayed minor clinical signs of disease (average clinical score of 1) and no animals had succumbed to disease prior to initiation of treatment. Mice received a human equivalent dose of either finafloxacin (37.5 mg/kg every 8 h) or co-trimoxazole (78 mg/kg every twelve hours) for 14 days. At the end of the treatment period, and in each of the subsequent 7 weeks, weekly culls (at days 15, 22, 29, 36, 43, 50, 57, and 66 days post infection) were performed (n=5 per cull group) Here, organs were harvested for bacterial enumeration and samples processed. Animals were weighed daily and monitored twice daily for clinical signs of disease and survival for 66 days.

**Figure 1:**
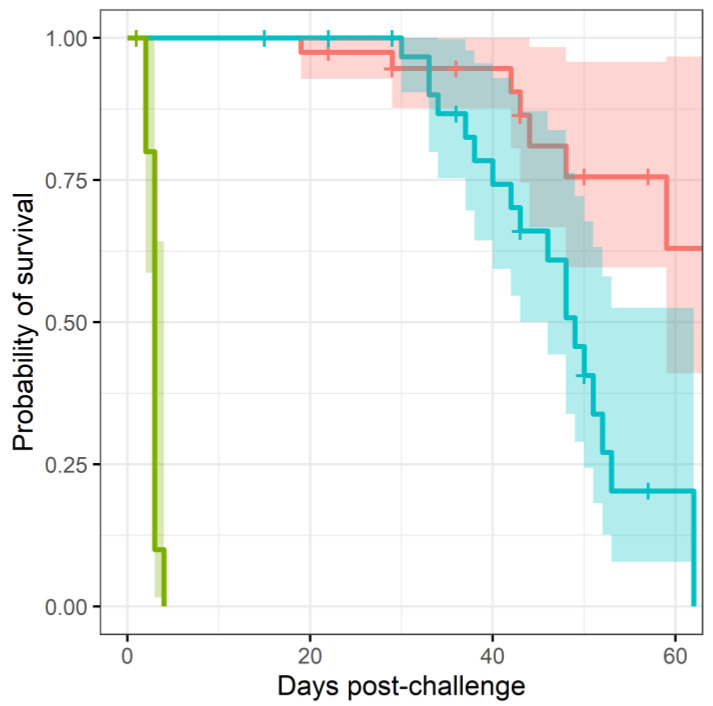
Survival characteristics of mice infected with *B. pseudomallei* and treated with antibiotics. This plot shows the probability of mice reaching a humane endpoint when treated with finafloxacin (red line), co-trimoxazole (blue line) or untreated and vehicle treated (green line). Mice were infected with a mean retained dose of 142 CFU of *B. pseudomallei* by the inhalational route and treated with finafloxacin (37.5 mg/kg) every 8 h or co-trimoxazole (78 mg/kg) every 12 h, orally, for 14 days, initiated at 24 h post-challenge. A group of animals were challenged and treated with the vehicle every 8 h. The crosses indicate the scheduled cull points. The shaded regions indicate the 95% confidence region. Supplemental Figure S1 is a diagnostic plot for this analysis.

**Figure 2:**
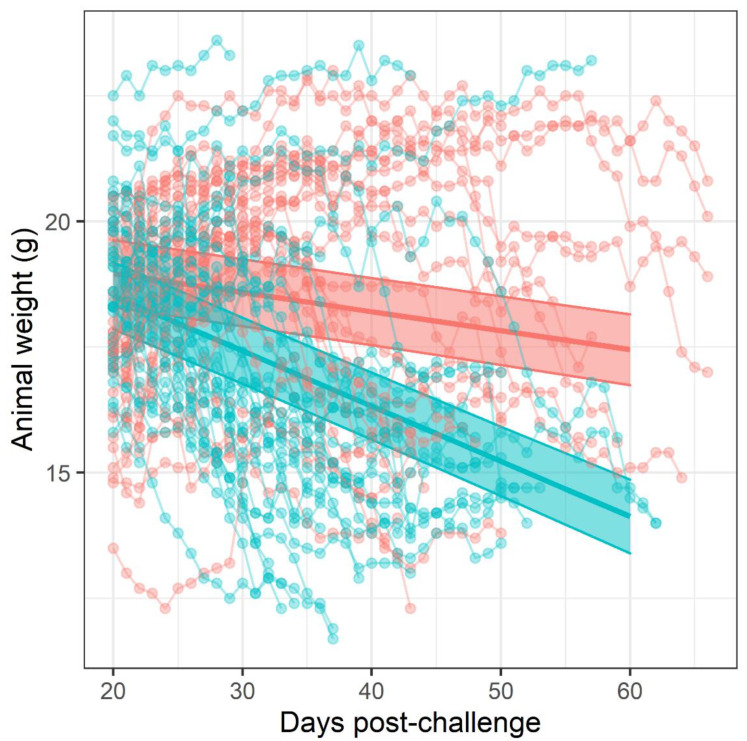
The weight loss profile recorded following antibiotic treatment. Mice were infected with a mean retained dose of 142 CFU of *B. pseudomallei* by the inhalational route and treated with finafloxacin (37.5 mg/kg) every 8 h or co-trimoxazole (78 mg/kg) every 12 h, orally, for 14 days, initiated at 24 h post-challenge. A group of animals were challenged and treated with the vehicle every 8 h. Mice were weighed daily throughout the study. The shaded area represents the 95% prediction interval from day 20 post-challenge, the dots represent the original data points. Red–mice treated with finafloxacin, blue–mice treated with co-trimoxazole. Supplemental Figure S2 is a diagnostic plot for this analysis.

**Figure 3:**
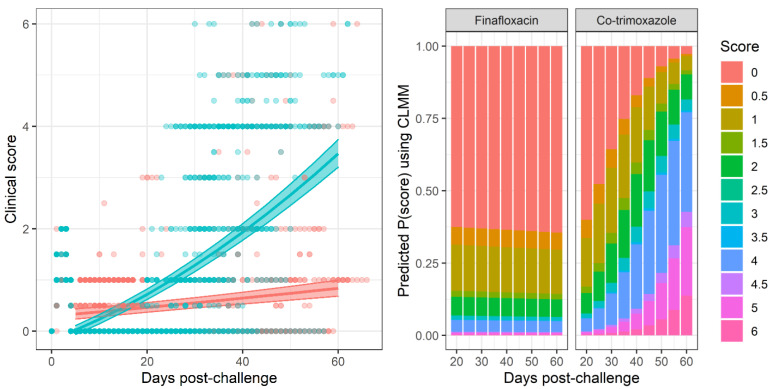
The clinical scores recorded following antibiotic treatment. Mice were infected with a mean retained dose of 142 CFU of *B. pseudomallei* by the inhalational route and treated with finafloxacin (37.5 mg/kg) every 8 h or co-trimoxazole (78 mg/kg) every 12 h, orally, for 14 days, initiated at 24 h post-challenge. A group of animals were challenged and treated with the vehicle every 8 h. Clinical signs of disease were recorded twice daily throughout the study and are shown in (**left**) using the mixed linear model prediction (i.e., the likelihood of each clinical score as interpreted by the model). The shaded area represents the 95% prediction interval, the dots represent the original data points. Red–mice treated with finafloxacin, blue–mice treated with co-trimoxazole. (**right**) shows the predictions of a cumulative link model in heat map form showing the density of the scores associated with the time point. Supplemental Figures S3 and S4 are diagnostic plots for this analysis.

More mice treated with co-trimoxazole succumbed to resurgent disease (p<0.0025, using a Cox regression model), apparent by the number of mice reaching their humane end point (the point where the animals are culled because they are not expected to recover) (Figure 1). The difference in mortality also translated into an observable difference in both weight loss (Figure 2) and clinical signs of disease (Figure 3). Statistical modelling was performed on the body weight and clinical score datasets from day 20 post-challenge, to capture the trajectory of the re-emerging infection. Two complementary statistical techniques were used to analyse the data. A general linear mixed model and a cumulative link mixed model were used to model changes in animal weights and clinical signs (respectively) over time. In both cases, a random intercept mixed model was used due to repeated measures. During treatment and up to day 20 post-challenge, the mice demonstrated few observable signs of infection. These statistical models indicated that finafloxacin was superior to co-trimoxazole in protecting against weight loss and the development of clinical signs of infection (p<0.001, value for interaction antibiotic and time). A generalised linear mixed model of the clinical scores dataset was also fitted, but showed some deviation from the model assumptions (Supplemental Figure S3). For this reason, a second model was constructed with fewer test assumptions. This model found similarly significant results; that finafloxacin was superior to co-trimoxazole in protecting against the development of clinical signs of infection (p<0.001, value for interaction antibiotic and time).

### 2.2. Bacteria Rapidly Re-Emerges within Organs

**Figure 4:**
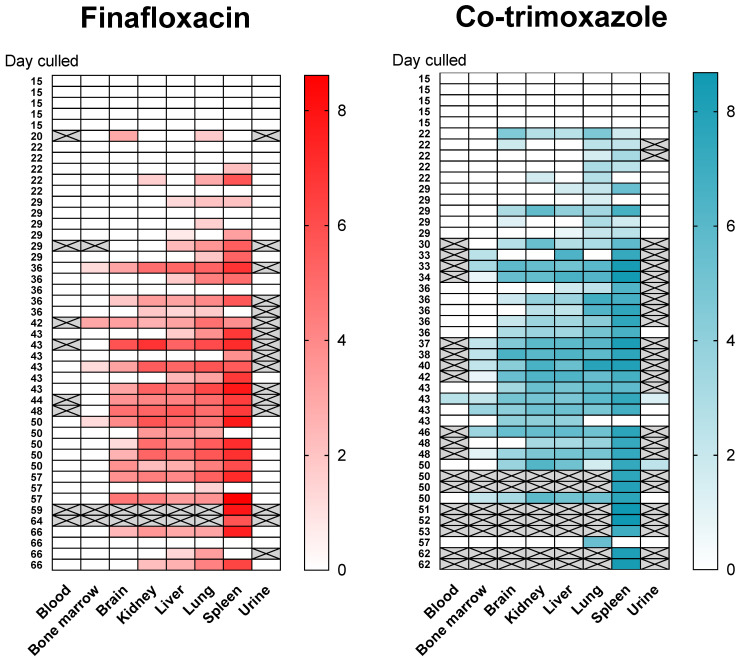
The bacterial burden in mice. Mice were infected with a mean retained dose of 142 CFU of *B. pseudomallei* by the inhalational route and treated with finafloxacin (37.5 mg/kg) every 8 h or co-trimoxazole (78 mg/kg) every 12 h, orally, for 14 days, initiated at 24 h post-challenge. A group of animals were challenged and treated with the vehicle every 8 h. A cross indicates data not collected. The data for some mice are not shown if they had reached a humane endpoint and data was not collected in its totality. Additionally it was not possible to obtain urine, blood and a single lung from all mice. It is important to note that fewer mice reached the end of the experiment when treated with co-trimoxazole and therefore less mice were available at the later time points for bacterial analysis. The colour intensity relates to the bacterial burden (CFU) transformed to the logarithm of 10 (i.e., 1=10 and $8=10^{8}$). Each row is a mouse and the time of cull (in days) is given on the y-axis. (**left**) mice treated with finafloxacin and (**right**) co-trimoxazole.

Following infection (and consistent with other published studies) the bacteria replicated rapidly within the primary loci of infection (the lungs) and disseminated to all organs evaluated within 4 days (Supplemental Figure S5). The mice had high bacterial loads in the lungs, ranging from $1.71\times 10^{5}$ to $1.04\times 10^{6}$ CFU/g at the onset of treatment. At the end of both 14 day treatment regimens, the bacterial load was reduced to below the limit of detection (~5 CFU based on duplicate plating of 1/20th of the neat organ homogenate) in the lungs, liver, spleen, brain, kidney and blood. Bacteria were not detected again until day 22 post-challenge (7 days post antibiotic cessation, Figure 4). A total of 65 mice were culled at scheduled time points and a further 25 mice were culled because they had reached their humane endpoint (7 mice treated with finafloxacin and 18 treated with co-trimoxazole). One way to understand where bacteria might re-emerge from may be to identify a single locus of infection at the time point when disease returns. Of the mice with a single focus of infection, 4 had bacteria colonising only the lung (2 treated with finafloxacin, 2 treated with co-trimoxazole) and 2 mice had bacteria colonizing only the spleen (both treated with finafloxacin). All but 5 of the animals that were colonised with bacteria (5 of 59, 4 treated with finafloxacin, 1 treated with co-trimoxazole), had *B. pseudomallei* in the lung; whereas, all but 10 had detectable bacteria in organs other than the spleen (10 of 59, 6 treated with finafloxacin, 4 treated with co-trimoxazole). There were no incidences of bacteria only isolated from the liver, kidneys and brain and not from the other organs. However, one mouse (which was treated with co-trimoxazole) had bacteria in the liver, kidneys and brain (but not the lung or spleen). This mouse was culled at 43 days post-challenge at a scheduled cull point. Collectively these data do not point to a specific location where bacteria were located and able to survive treatment.

No bacteria were detected in the bone marrow until day 29 post-challenge when two mice treated with co-trimoxazole were colonised. No bacteria were found in the blood until day 43 post-challenge, when a single mouse from each treatment group was colonised. Throughout the study only one mouse had *B. pseudomallei* in the blood (1 of 64 tested–blood was not taken from all animals) and sixteen mice had bacteria in the bone marrow (16 of 80 tested–bone marrow was not taken from all animals, 4 in the finafloxacin treated group and 12 in the co-trimoxazole treated group) and therefore the data could not be analysed statistically in the same way as for the other organs.

Investigation of the lymph nodes using bacteriological methods in mice is technically very challenging due to their small size and their integration into the lymph vessels and organs. For this reason, lymph nodes were investigated using histopathological techniques. Lymph nodes were extracted from animals culled at days 1, 15, 22 and 29 post-challenge and analysed for changes in structure or the presence of lesions caused by infection with *B. pseudomallei*. None of the lymph nodes analysed demonstrated any evidence of specific acute or chronic inflammatory changes associated with infection. Any non-specific changes observed were deemed consistent with active or reactive lymph nodes and were within the normal range (data not included).

**Figure 5:**
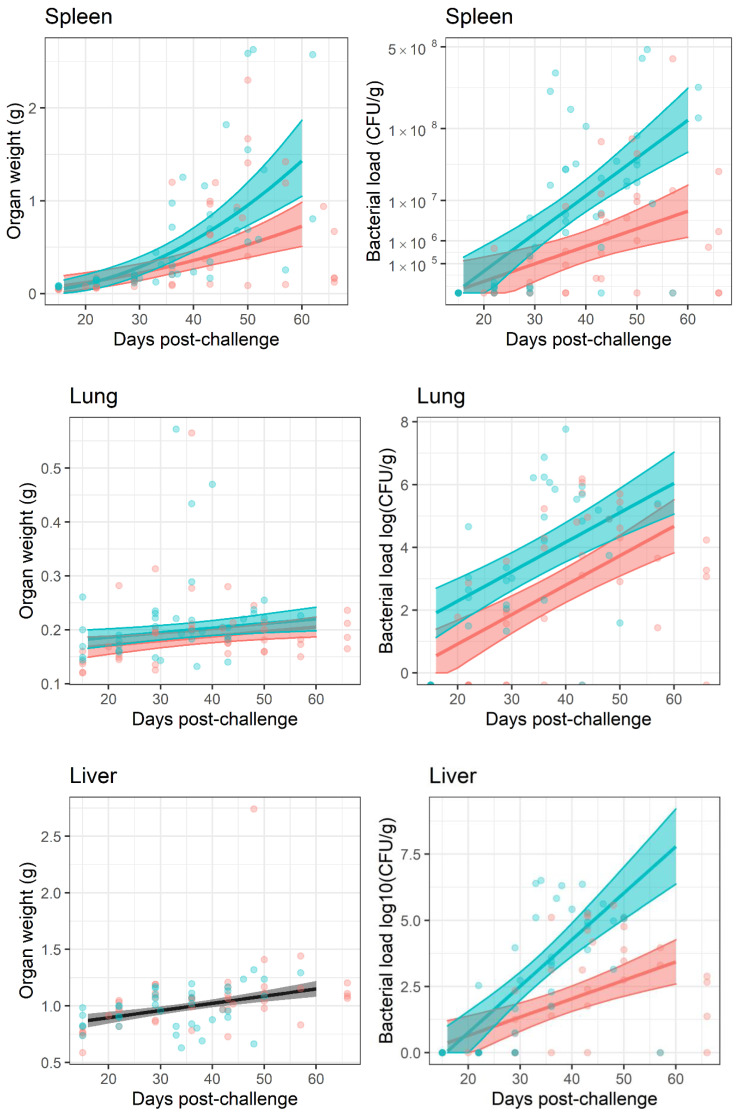

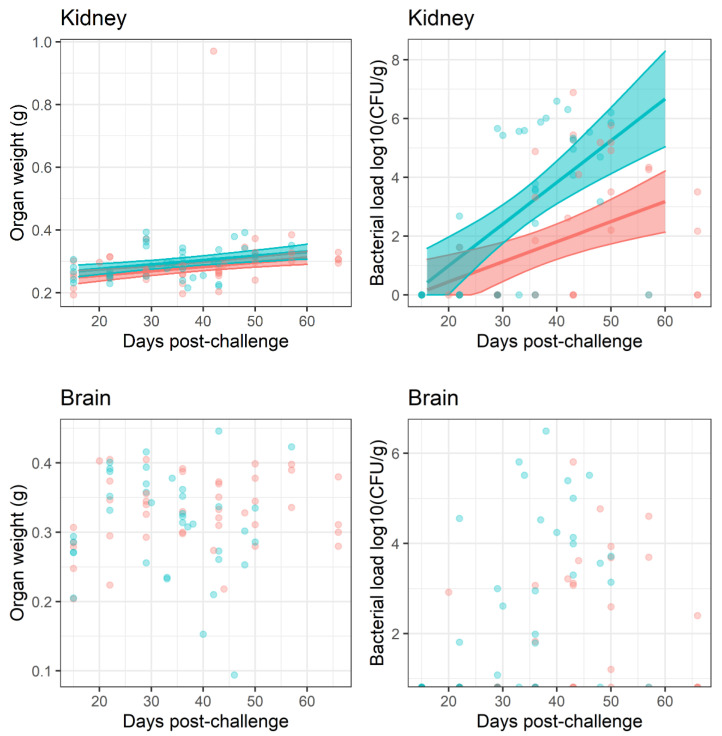
Comparison of the bacterial burden and organ weights of mice post-challenge. Mice were infected with a mean retained dose of 142 CFU of *B. pseudomallei* by the inhalational route and treated with finafloxacin (37.5 mg/kg) every 8 h or co-trimoxazole (78 mg/kg) every 12 h, orally, for 14 days, initiated at 24 h post-challenge. A group of animals were challenged and treated with the vehicle every 8 h. Each data point represents data from a single mouse. The shaded area represents the 95% prediction interval, the dots represent the original data points. Red–mice treated with finafloxacin, blue–mice treated with co-trimoxazole, grey–indicates overlapping data from both antibiotics. Data was split by organ: spleen, lung, liver, kidney and brain. Supplemental Figure S6 is a diagnostic plot for this analysis.

A gross effect of inflammation is an increase in organ weight. Organs were weighed post-mortem and the determined weight was used to normalise bacterial burden individually. This normalisation had little effect on the interpretation of the bacterial load data because organ weights ranged on a linear scale and bacterial burden on a logarithmic scale. The greatest difference in organ weight over the time course of the study was approximately 10-fold. This occurred in the spleen, which were colonised with a bacterial load ranging from 0 to $10^{8}$ CFU (Figure 5). Linear models were generated to interpret organ weight change data. These models indicated that the dataset provided strong evidence for organ weight increasing over time for the lungs, liver, spleen and kidneys (p<0.05, main effect of time) but not brain (p>0.05) (Figure 5). The models also suggested that increases in organ weight were reduced in animals treated with finafloxacin when compared to those treated with co-trimoxazole in the spleen, as the interaction with time (p<0.05, where the effect of treatment is greater later in the experiment) and in the lungs and kidneys as a main effect (p<0.05, the effect is consistent throughout the experiment); but not in the brain or liver (p>0.05).

Once the infection had re-established, the bacterial counts increased Figure 5. Bacterial load data was analysed by linear models for each organ and treatment, from day 20 post-challenge, when the bacteria were detected. Increasing titres of bacteria were observed in all organs with the exception of the brain (p<0.05, for the effect of time in all models). The models also indicated that finafloxacin restricted bacterial growth when compared to co-trimoxazole in the spleen, liver and kidneys, where the effect increased over time after infection (p<0.05, as an interaction) and the lung (p<0.05, as a main effect). There was no evidence for a treatment effect in the brain with regard to bacterial counts or organ weights.

### 2.3. No Evidence for the Development of Antibiotic Resistance Was Observed

Three isolates of *B. pseudomallei* were harvested from each mouse treated with finafloxacin or co-trimoxazole that survived until the end of the study. None of the isolates harvested showed an increase in MIC when compared to the strain of *B. pseudomallei* used for the bacterial challenge (2 µg/mL and 16 µg/mL for finafloxacin or co-trimoxazole respectively). This suggests that the relapse of infection was not due to the development of resistance during the study.

### 2.4. The Effect of Bacterial Re-Emergence on Inflammation

In addition to increases in organ weight, there are a variety of ways how inflammation can be phenotyped. This study used two immunological techniques to evaluate the progression of the innate immune response.

The presence, and therefore production and release, of inflammatory markers were measured using a commercial 23-plex Luminex assay. In a similar way to the bacterial load data, the cytokine concentrations were standardised against the weight of the organs. The concentrations of 23 cytokines were determined in the spleen, lungs and plasma, for the 64 mice culled over a period of 7 weeks.

**Figure 6:**
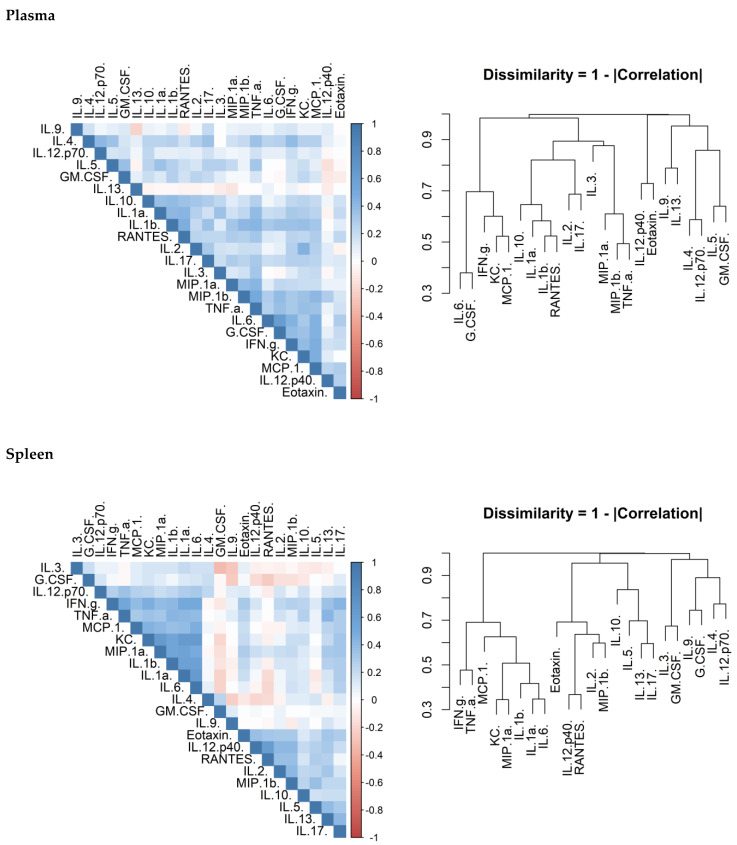

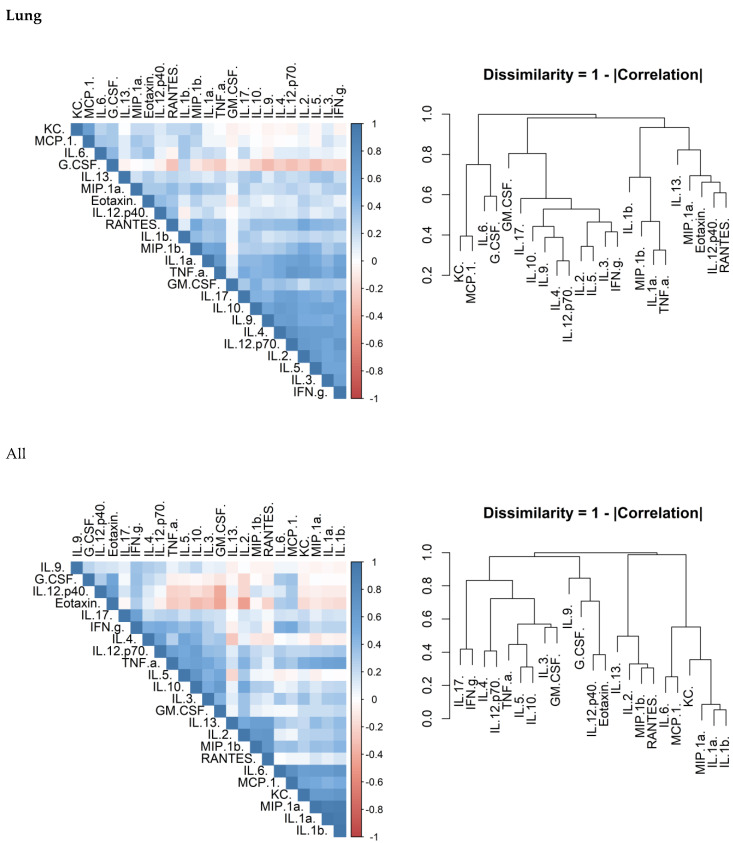
The relationship of cytokine concentration in animals treated with antibiotics. Correlation matrices detailing the relationship between the concentrations of different cytokine in different organs. Mice were infected with a mean retained dose of 142 CFU of *B. pseudomallei* by the inhalational route and treated with finafloxacin (37.5 mg/kg) every 8 h or co-trimoxazole (78 mg/kg) every 12 h, orally, for 14 days, initiated at 24 h post-challenge. The data was from both treatment groups combined. The order of the metric was derived based on cluster analysis. The colour density associated with each cell is the Pearson’s correlation mapped to the colour-scale left of the matrix. This data is also shown as dendrogram for dissimilarity (1 minus Pearson’s r divided by 2). In this way, the analytes have been clustered by similarity. This data was split by plasma, spleen, lung and expressed collectively.

There is considerable overlap in the systems that control cytokine production and release. Correlation matrices were produced to confirm that this was true of the data generated in this study. There was a correlation between cytokines within each of the investigated organs (Figure 6). Another observation was that the concentrations of these cytokines appeared to cluster.

To explore these data further, a supervised machine learning approach was taken. Multiple common classification algorithms were used to classify the effect of treatment with finafloxacin and co-trimoxazole for the Luminex array data from each organ (Supplemental Table S1). We observed poor classification performance in all models across all organs, suggesting there was not a strong signature in the data indicative of treatment type. However, this is perhaps not unexpected as whilst the entire dataset enabled the classification model to be built, it is confounded by the data coming from different time points and severities in animal condition. Further, the dataset (74 dependant variables per organ across both cohorts) is relatively small considering the complexity of the metadata (time, disease severity). We used the random forest model further and considered which cytokines were important for predicting treatment success (Supplemental Figure S8). This analysis identified eotaxin but only for an overall (time-independent) difference (p=0.009, Supplemental Figure S9).

### 2.5. The Dynamics of Leukocyte Densities

**Figure 7:**
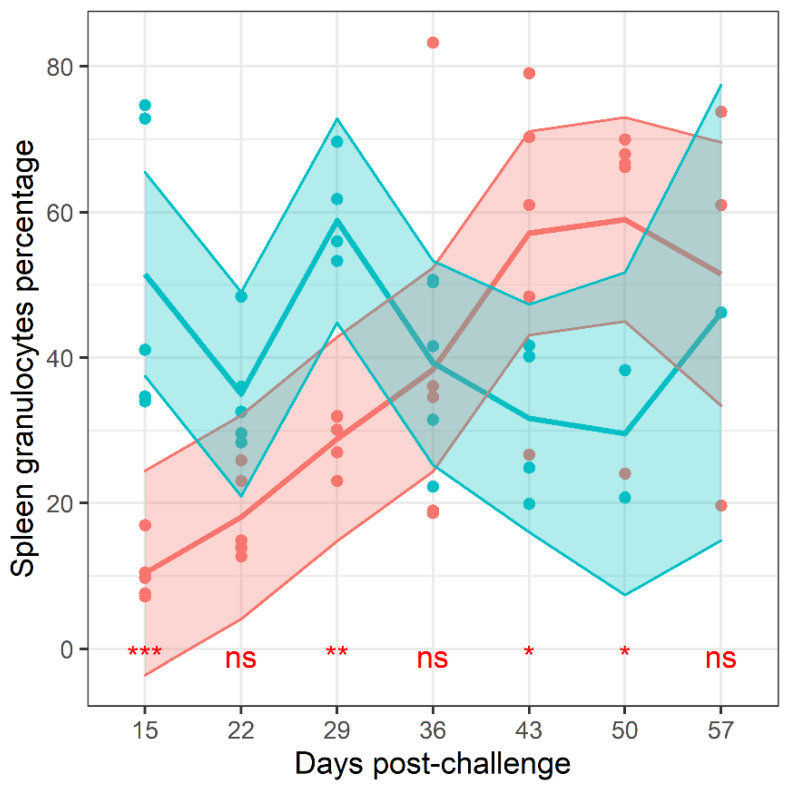
The percentage of granulocytes in the spleen following treatment with antibiotics. Flow cytometry was used to determine the proportion of different immune cell types and markers in the lung, plasma and spleen tissues over time. Red–mice treated with finafloxacin, blue–mice treated with co-trimoxazole, grey–indicates overlapping data from both antibiotics. Across the bottom of the plot are markers to denote the likelihood of the difference between treatment groups occurring randomly: ns = no significance, * p<0.05, ** p<0.01 and *** p<0.001.

Flow cytometry was used to estimate the relative proportions of different leukocytes in the samples collected throughout the study. The gating strategy first identified lymphocytes and granulocytes from the forward, side scatter plot. Neutrophils and macrophages were also independently identified from the leukocyte population using antibodies known to bind to the neutrophil specific antigen, Ly6-G, and CD14. Granulocytes were further divided into those expressing MHC class II and CD64. These cell proportions were analysed by a general linear model using time and treatment as explanatory variables. The proportion of many (7 of 13) of the populations of cells changed over time (Supplemental Table S2). These included CD64^+^ granulocytes in the lung (p=0.013), neutrophils in the spleen (p<0.013), neutrophils, granulocytes and lymphocytes in the blood (all p<0.013) and MHCII positive granulocytes (p=0.026). Only the splenic granulocyte proportion exhibited treatment-based altered change over time (p<0.013, interaction) (Figure 7). There was no evidence for treatment effects on the proportion of any other measured cell population.

## 3. Discussion

In this study, the re-emergence of *B. pseudomallei* following cessation of antibiotics is investigated using an inhalational mouse model of infection. It is apparent from the protection offered, the development of clinical signs of disease, bodyweight loss and bacterial load, that finafloxacin was more effective at controlling infection when compared to co-trimoxazole, a finding that has been previously determined [31]. This work builds on published data by providing a time point-based analysis of the relapse of infection associated with cessation of treatment. This study demonstrates that the greater protective capability of finafloxacin correlates with a better control of bacterial growth later on in the infection.

Both antibiotic regimens were able to reduce the bacterial load to below detectable levels within the lungs, liver, spleen, kidney and brain in the course of a 14 day treatment period. This aligned with previous studies using this model [31]. Despite this, the infection re-emerged following cessation of treatment. There was no evidence to demonstrate bacteria re-emerging from a single location, although the most common location to first isolate bacteria was the lungs, followed by the spleen. These observations can be interpreted in a variety of ways. Firstly, it is possible that bacteria were eradicated from the organs under investigation and re-emerged from a location that was not evaluated or accessible perhaps in an endosome (like mycobacteria) or walled-off in a granuloma. Secondly, bacteria surviving treatment may have converted into a non-culturable, persister-like or differentially culturable state [35,36,37]. These bacteria may have reverted to actively growing bacteria following cessation of antibiotics. Further study is required to confirm these hypotheses. The lymphatic system and the gastrointestinal tract could also be potential refuges. There was no evidence of infection in the lymph nodes evaluated immediately after cessation of antibiotics. It is also known that *B. pseudomallei* can be isolated from the stool of human patients [38]. Moreover, multiple previous studies have shown that experimental models are sensitive to *B. pseudomallei* challenge via the gastro-intestinal tract [39,40,41]. However, there was no evidence of the mesenteric lymph node being infected following cessation of antibiotics in this study.

Inflammation is the host response to pathogen and damage associated molecular patterns (PAMPs and DAMPs) and is often correlated directly to the quantity of these signals. An investigation to compare the effects of two different antibiotics in altering the concentration of inflammatory markers was performed. These concentrations can result in different effector responses and the extent of this is illustrated by the differences between the T_h_1, T_h_2 and T_h_17 responses. Preliminary investigation in this study suggests that there are correlations between cytokines (confirming collective expression control) and also cytokine clusters. In the lungs and plasma, chemokines and IL-1 correlated together. T_h_1, T_h_2 and T_h_17 biasing cytokines correlated well together in the lung demonstrating that a broad response was mounted. The response in the spleen was similar; however, there was no or little evidence for a T_h_2 response. The data was comparable to our previous explorations of cytokine levels in this model [42]. As a lymphatic organ, it is not surprising that there was a stronger immune bias towards cytokines associated with the T_h_1 and T_h_17 in the spleen. These responses are more associated with targeting bacterial infection [43]. The presence of T_h_2 cytokines in the lung but not the spleen is more difficult to explain. It may be that these cytokines are required to maintain tissue integrity for lung function through the presence of alternatively activated macrophages.

As the bacterial load within tissues was shown to be affected by the two antibiotic treatments, and that inflammation correlated with bacterial load, investigation of the inflammatory responses at each time point was performed. A random forest classifier technique was used to investigate the treatment-based differences in cytokine concentration. The time following infection was not included in these statistical models, so that this comparison was less about the magnitude of response and more about the difference in profile. The best biomarker for differentiating between the treatments was the concentration of eotaxin in the lung. Throughout the study, eotaxin levels were higher in the animals treated with co-trimoxazole. It was also notable that eotaxin levels did not correlate well with other cytokines. Eotaxin is known to be chemotactic for eosinophils and, in humans, there are three known family members: CCL11 (eotaxin-1), CCL24 (eotaxin-2) and CCL26 (eotaxin-3) [44]. The Luminex kit used in this study did not differentiate between subtypes. Eosinophils are a class of granulocyte that are traditionally associated with allergy and anti-parasitic processes, although it is known that eosinophils can also have a phagocytic role in bacterial removal [45]. There was no evidence for the cytokines that regulate eosinophil maturation and release (i.e., IL-5, IL-5, and GM-CSF) being differentially regulated between the treatment groups. Altered frequencies of eosinophils may also play a role in the re-emergence of infection, where one antibiotic can protect and another cannot. In the Common Marmoset model, a systemic increase in eosinophil levels was observed following oral infection with *B. pseudomallei*. It has been suggested that this may be due to gastro-intestinal (GI) disturbance, bacteria migrating from the gut to the lungs through the lymphatic system with the eosinophils. One interpretation is that, in this study, the bacteria were residing in the GI tract and when systemic antibiotic levels were reduced, they remerged from the gut to the other organs. Further study to confirm this is required, however determining the bacterial load in the (GI) tract is complicated by the colonisation of commensal gut bacteria.

The proportion of several leukocyte populations were also determined during this experiment. Dynamic changes in the frequencies of leukocytes were expected as the infection re-established. There was only evidence for treatment differences for a single cell population, the level of granulocytes in the spleen which was lower in the mice treated with finafloxacin but subsequently increased to a proportion greater than those measured in the spleens of animals treated with co-trimoxazole. These cells comprise neutrophils, eosinophils and basophils. We found no evidence for differences in neutrophil proportion (a subset of granulocytes). This suggests that the whole pantheon of granulocytes are increasing in a frequency relative to lymphocytes. Neutrophils are most associated with the host response towards *B. pseudomallei* [46] and as such it is reasonable to assume that their recruitment would be prioritised over other granulocytes. This recruitment of neutrophils has been shown to be high in mice infected with *B. pseudomallei* [47].

One potential issue to consider when comparing antibiotics in vivo is whether any observed differences may be due to the development of antibiotic resistance. *B. pseudomallei* has multiple mechanisms of antibiotic resistance that can differ between isolates, indicating how capable this pathogen is at responding to different antibiotic treatment [48]. In the study described here, the antibiotic sensitivity of bacterial isolates harvested from surviving animals at the end of the study were evaluated using an MIC assay. No difference in antibiotic sensitivity was identified.

## 4. Materials and Methods

### 4.1. Bacteria

*B. pseudomallei* strain K96243 was prepared by adding 10 µL of a frozen bacterial stock to 75 mL of Luria Bertani broth (L-broth) and incubating at 37 °C with shaking at 180 rpm for 18 h. The culture was adjusted to an optical density of 0.34 at 590 nm, to obtain approximately $1\times 10^{8}\;\mathrm{CFU}/\mathrm{mL}$. The culture was diluted 1 in 10 for use in the efficacy study.

### 4.2. Mice

Animal studies were performed in accordance with the United Kingdom Scientific Procedures Act (Animals) 1986 and the United Kingdom Codes of Practice for the Housing and Care of Animals Used in Scientific Procedures, 1989. Investigations involving animals were carried out according to the requirements of the UK Animal (Scientific Procedures) Act 1986 under the authority of a Project Licence P1D46FB69 granted by the UK Home Office. This project licence was approved. Female Balb/c mice (Charles River Laboratories, Harlow, UK) aged 8–10 weeks were housed cages of 5 within an ACDP Containment Level 3 laboratory. Animals were randomly assigned to groups prior to the bacterial challenge using random number tables. Where a substantial number of animals had succumbed to infection within a cage (i.e., 4 or 5 of 5 animals), the group to be culled in the subsequent time point was substituted for this one. This only occurred for the penultimate time point in the mice treated with co-trimoxazole. This has the potential for a “cage effect”, however, these effects would be spread randomly across the time course. Moreover, we have seen little evidence for “cage effect” being a meaningful measure of outcome in our experiments. Mice had free access to water and rodent diet (Harlan Teklad, Blackthorn, UK) and underwent a 7 day acclimatization period before any procedures were performed.

### 4.3. Antibiotics

For use in animals, finafloxacin powder (provided my MerLion, Berlin, Germany) was diluted to 15 mg/mL in Tris buffered saline and an oral suspension of co-trimoxazole (Aspen, Dublin, Ireland) was diluted to 78 mg/mL in Phosphate Buffered Saline. MICs on recovered bacteria were carried out using E tests, finafloxacin (AB Biodisk, Solna, Sweden) and co-trimoxazole (Biomerieux, Basingstoke, UK) as per the manufacturer’s instructions.

### 4.4. Efficacy Study

Animals were challenged with an aerosol of B. pseudoma*llei*. Mice were restrained within a nose only exposure tube and placed within an exposure chamber, which was connected to a Collison three jet nebulizer by a spray tube and controlled using an AeroMP apparatus (Biaera Technologies, Hagerstown, US). Fifteen millilitres of bacteria were placed into the nebulizer and mice exposed for 10 min to a dynamic aerosol conditioned in an AeroMP apparatus. The aerosol stream was maintained at 50–55% relative humidity and 22 °C. The concentration of *B. pseudomallei* in the aerosol was determined by recovering samples from the exposure chamber using an All Glass Impinger operating at 12 L/min, containing 10 mL of sterile PBS. Impinger samples were plated out onto L-agar for bacterial enumeration, and the retained dose of bacteria that mice received in each run was calculated by applying the Guyton formula [49]. It was assumed that each mouse retained 40% of the organisms that were inhaled [50].

For antibiotic treatment mice weights were approximated to 20 g. Treatment was initiated at 24 h post-challenge, and continued for 14 days; groups of mice were administered with finafloxacin (37.5 mg/kg) in a 50 µL oral dose via pipette every 8 h (each dose containing 0.75 mg of finafloxacin) or co-trimoxazole (78 mg/kg) in a 50 µL oral dose via pipette every 12 h (each dose containing 1.56 mg of co-trimoxazole). Mice were dosed with 2.25 mg of finafloxacin, or 3.12 mg of co-trimoxazole per 24 h. Control groups of infected mice were administered 50 µL of the vehicle (consisting of Tris Buffer, sodium hydroxide and hydrochloric acid, adjusted to pH8) orally via pipette every 8 h. Mice were weighed daily and observed twice daily for clinical signs of disease for 66 days when the experiment was terminated. Five mice per treatment group were culled at cessation of antibiotic therapy (day 15 post–challenge) and then at weekly intervals (days 21, 28, 35, 42, 49, 56, 66 post–challenge). In addition, 5 naïve mice were culled to determine baseline levels for the immunoassays and 5 mice were infected and culled at treatment initiation. Post mortems were performed on all mice, and the spleen, liver, lungs, kidney, brain and urine harvested, weighed and tissue samples collected and selected samples underwent histopathological analysis. Bone marrow and blood was harvested for bacterial enumeration and plasma removed for immune analysis. For a neat suspension approximately one fifth of the organs were plated out, therefore the lower limit for detection was determined as 5 CFU.

The remaining organs were homogenised in 1 mL of PBS. A 10-fold serial dilution was performed and 100 µL aliquots were plated onto L-agar in duplicate. The agar plates were incubated for 48 h at 37 °C and bacteria were enumerated to determine the bacterial load in the organs. The remaining homogenate was placed into 10 mL of L-broth and incubated at 37 °C for 7 days. For organs that were clear by plate count, a 10 µL loop of the relevant incubated homogenate was streaked onto L-agar plates and incubated at 37 °C for a further 4 days, after which the visual presence of *B. pseudomallei* was determined.

### 4.5. Histopathological Analysis

Samples fixed in buffered formalin were embedded in paraffin wax using routine processing methods. Lymph nodes were harvested and four micron thick sections were stained with haematoxylin and eosin for histological examination.

### 4.6. Luminex Assay

The Bio-plex Pro Mouse Cytokine 23-plex assay (Bio-Rad, Hercules, US) was used to look at differences in the level of a panel of cytokines. This was performed on 50 µL of plasma, spleen or lung homogenate harvested at the time points detailed above. The kit was used as per manufacturer’s instructions. The final extrapolated concentration was taken from the analysis software. The readings below the limit of quantification were given a value of 0.9 of the minimum and the readings above given the maximum +1. Only the cytokines from mice culled at the predetermined time points were analysed using Luminex.

### 4.7. Flow Cytometry

A mixture of 50 µL of spleen homogenate, 100 µL of lung homogenate or 50 µL of blood were resuspended in 1.9 mL of red cell lysis buffer, and incubated at room temperature for 10 min. All samples were centrifuged at 400×g for 5 min, washed with 1 mL PBS and centrifuged. Samples were resuspended in 100 µL flow buffer (2% *w*/*v* foetal bovine serum in PBS) with 3 µL TruStain FcX (anti-mouse CD16/32). After a 5 min incubation at room temperature, 50 µL of antibody staining master mix was added, and the cells incubated for 40 min (at room temperature). After staining, the samples were washed with 1 mL flow buffer, centrifuged, and resuspended in 200 µL of 4% paraformaldehyde (PFA). Single colour samples were also prepared and used as compensation controls to calculate and compensate for fluorescence cross over between fluorophores. Samples were run on a FACS Canto II using Diva 6.0 control software. Data analysis was completed in FlowJo version 10.4.

The stain included antibodies against CD45 (Clone = 30-F11, APC/Fire 750, 3 µL per sample, pan-leukocyte), Ly6G (Clone = 1A8, 2.3 µL per sample, Brilliant Violet 510, neutrophil), CD14 (Clone = Sa14-2, 3 µL per sample, APC, monocytes), CD80 (Clone = 16-10A1, 4 µL per sample, AlexaFluor™ 488, activation marker), CD86 (Clone = GL-1, 5 µL per sample, PerCP-Cy5.5, activation marker), CD64 (Clone = X54-5/7.1, 1.5 µL per sample, Brilliant Violet 421, activation marker) and MHC class II (Clone = M1/42, 3 µL per sample, PE, activation marker). Sequential gating was used to estimate cell proportions (supplemental Figure S10). Cells were first gated using forward scatter (FSC) vs. side scatter (SSC), followed by double reduction using SSC-width vs. SSC-height and FSC-width vs. FSC. Leukocytes we then selected using SSC vs. CD45 gate. Macrophages and neutrophils were selected using a CD14 vs. Ly6G gate. Granulocytes were also selected (independently from macrophages and neutrophils) from the leukocyte population, using FSC vs. SSC and categorized as SSC^high^ FSC^high^. Activation on each cell subtype was then measured using a histogram of the cell population CD64, CD86 and MHC II graphs.

### 4.8. Statistics

Statistical modelling and some graphs were prepared using the R statistical computing language (version 3.6.1) and R studio (version 1.2.1335). Additional packages were also installed for specific analyses and were all appropriate for this version of R. Other graphs were prepared using the software Graphpad PRISM v8.0.

#### 4.8.1. Survival Analysis

Cox proportional hazards regression (Cox-PH) was used to assess the statistical significance when comparing the survival rates of mice treated with finafloxacin or co-trimoxazole. This is an appropriate semi-parametric model for a hazard using time to event data with right censorship. The Cox regression model assumes proportional hazards with a linear relationship between the log of the hazard and each variable and are susceptible to influential outliers. Checking these assumptions is performed using specifically defined residuals for Cox regression models and testing. First, Schoenfeild residuals were used to assess the assumption of proportional hazards. Further, where appropriate Martingale residuals to assess the functional form of continuous covariates, and deviance residuals are used to identify influential outliers. The Schoenfeild individual test was used to determine the statistical significance of trends observed in the Schoenfeild residuals, indicative of a violation of the assumption of proportional hazards. More information about the assumptions of Cox models and definitions for these residuals can be found at [51]. Post hoc statistical tests were used to assess the significance of the different proportional hazard in each group. Kaplan-Meyer curves were used to visualise the survival within each group with 95% confidence intervals.

#### 4.8.2. Analysis of Body Weight over Time

General linear mixed models (GLMM) were used to assess the trend in weight over time in each treatment group. Mice were each weighed at specific time points post-challenge and therefore individual weight measurements were not independent but part of a time-series for each mouse. Therefore, a mixed-modelling approach was used to account for repeated measures and a random intercept but not random slope was selected using the Akaike’s information criterion (AIC). We additionally considered including the mouse group (those housed in the same cage for the experiment) for the random model, but this was not found to improve fit (using AIC). The initial fixed model included the days post-challenge, the antibiotic detail and the interaction of the two. The fixed model was refined by iterative removal of the least significant (Wald’s Chi^2^ test) and highest order predictor until all met the *p* < 0.05 significance threshold. The assumptions of the model including linearity of response, normality of residuals and homoscedasticity were checked initially and at each stage of model refinement using appropriate residual plots (normal Q-Q plot and residuals against fitted values using deviance residuals). Once a final model had been achieved predictions and post hoc statistical tests (adjusted for multiple comparisons using Tukey’s method) were generated.

#### 4.8.3. Analysis of Clinical Scores over Time

In addition to mouse weight a clinical score of disease was attributed to each mouse at specific intervals to record their health status. This again generated repeated measures for each mouse so mixed models were considered. However, this score is also a discrete-ordinal variable and not a continuous measure. A cumulative link mixed model (CLMM) was used with a random intercept for mice and where the response was the ordinal variable clinical score. The main assumption of cumulative link models is that of proportional odds, for each ordinal level the change by groups to the estimated proportions is uniform. This assumption is rarely held by real data and can be violated in two ways by nominal and scale effects tied to each predictor. These can be tested statistically and violations of these assumptions can be addressed by modification to the CLM’s threshold function or by inclusion of scale or nominal effect terms in the model at the cost of model sensitivity [52]. Further, the model assumes linearity and normality of response, which can be assessed using surrogate residuals (note the ordinal nature of the response variable does not allow for easy analysis of real residuals). The initial fixed model included days post-challenge, the antibiotic details and the interaction of the two. The fixed model was refined by iterative removal of the least significant (Wald’s Chi^2^ test) and highest order predictor until all met the *p* < 0.05 significance threshold. The assumptions of the model were checked initially and at each stage of model refinement using appropriate residual plots. Once a final model had been achieved, predictions and post hoc statistical tests (adjusted for multiple comparisons using Tukey’s method) were generated. Predictions were plotted using stacked-bar plots for the predicted proportions of each

A generalised linear mixed model was performed, using a method similar to that described for mouse weight. Whilst this model is not ideal as the response is not continuous, violating an assumption of the model, it does offer an alternative for visualisation where a predicted mean score can be plotted.

#### 4.8.4. Analysis of Organ Weights and Bacterial Load

For each organ, generalised linear models (GLM) were fitted for organ weight and bacterial load. The assumptions of each model were checked using appropriate residual plots and adjustments were made to improve the fit if necessary. Initial models included days post-challenge, treatment group and the interaction between the two. The models were iteratively refined by removal of the least-significant highest order predictor until all predictors were estimated to be *p* < 0.05 significant using Wald’s Chi^2^ test. Final models were used to generate predictions and for post hoc tests (adjusted for multiple comparisons using Tukey’s method).

#### 4.8.5. Analysis of Cytokine Titres

Correlation heat maps were generated to visualise which cytokines in each tissue correlated to each other using Pearson’s correlation coefficient. Whilst Pearson’s assumes linearity of the covariance, the rank based methods (Spearman’s and Kendall’s) are under-sensitive with low sample sizes where there are linear, or close to linear relationships. The disadvantage of using Pearson’s is falsely ignoring potential correlations where the relationship is monotonic but far from linear. Hierarchical clustering was used on the dissimilarity (defined as 1–the absolute of the correlation coefficient) and represented as a dendrogram.

Classification models were then generated for each treatment group using the cytokine array data, to see if differences in the cytokine profile could reliably be used to distinguish between the treatments. Due to the small scale of the data it was only split between testing and training sets as part of a K-fold strategy (using all data for both) with 5 groups, the data was also stratified by time-bins and by treatment. Random forest, support vector machines, Gaussian process and logistic regression were attempted and the performance of each model evaluated using Cohen’s Kappa. Appropriate methods for each model were used to assess significance of predictors.

#### 4.8.6. Analysis of Flow Cytometry Data

Generalised linear models were used to assess the proportion of specific cell types as recorded by flow cytometry. Measurements were taken at specific intervals and time was discretised to these specific intervals. The initial model included treatment group, time-interval and the interaction of the two. The model was refined by iterative removal of least significant variable starting with interactions (using F-test). The assumptions were checked by analysis of residuals for the initial model and each subsequent refinement. Once a final model had been selected (all variables meeting *p* < 0.05 significance) then the model was used to generate predictions for plotting and for post hoc statistical tests (adjusted for multiple comparisons using Tukey’s method).

## 5. Conclusions

To summarise, we have utilised two immunological methods that suggest that the presence and or activity of eosinophils may correlate with a more effective antibiotic. Firstly, we found greater eotaxin titres in the lungs of the finafloxacin treated mice. Secondly, we found that the granulocyte concentrations increased in the spleens of mice treated with finafloxacin. This was surprising as it was expected that the neutrophil population would increase more rapidly than the other granulocytes (traditionally comprising of eosinophils and mast cells. The data presented here indicates that further research is needed to establish the role of these cells in melioidosis. There is still much more to understand about where there bacteria are able to reside during antibiotic treatment and the specific nuances of disease relapse.

## Data Availability

Analysis is available on request.

## Supplementary Materials

The following supporting information can be downloaded at: https://www.mdpi.com/article/10.3390/antibiotics11101442/s1.
