# Supplementary material for: An Investigation into the Re-Emergence of Disease Following Cessation of Antibiotic Treatment in Balb/c Mice Infected with Inhalational Burkholderia pseudomallei
Source: Antibiotics (Basel). 2022 Oct 20;11(10):1442. doi: 10.3390/antibiotics11101442 (PMC9598772; doi:10.3390/antibiotics11101442)
Supplement: Supplementary file 1 [file antibiotics-11-01442-s001.zip › antibiotics-1947237-supplementary.pdf]

antibiotics

MDPI

Supplemental data

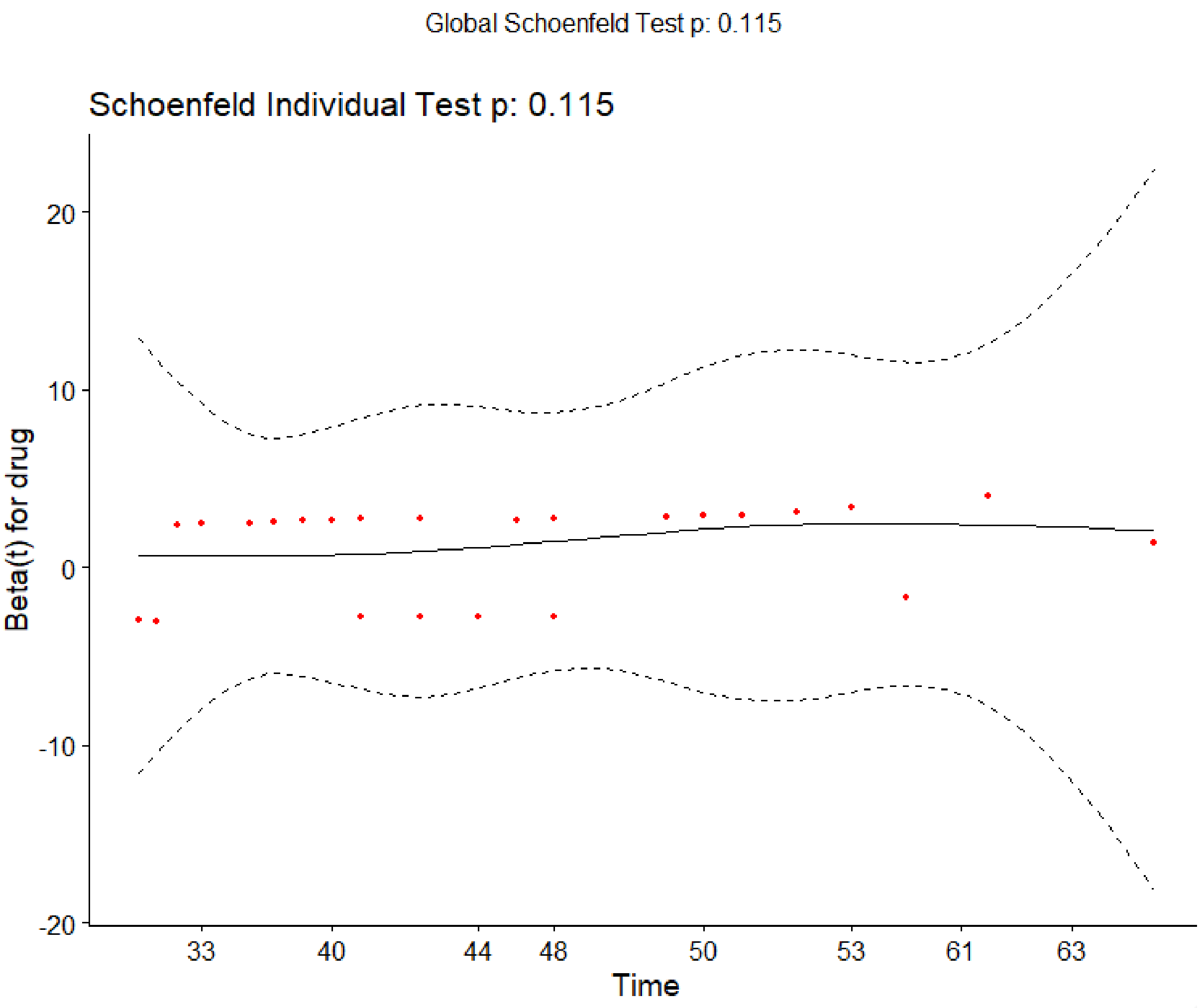

Supplemental Figure S1: Diagnostic plot for the survival analysis (Figure S1).

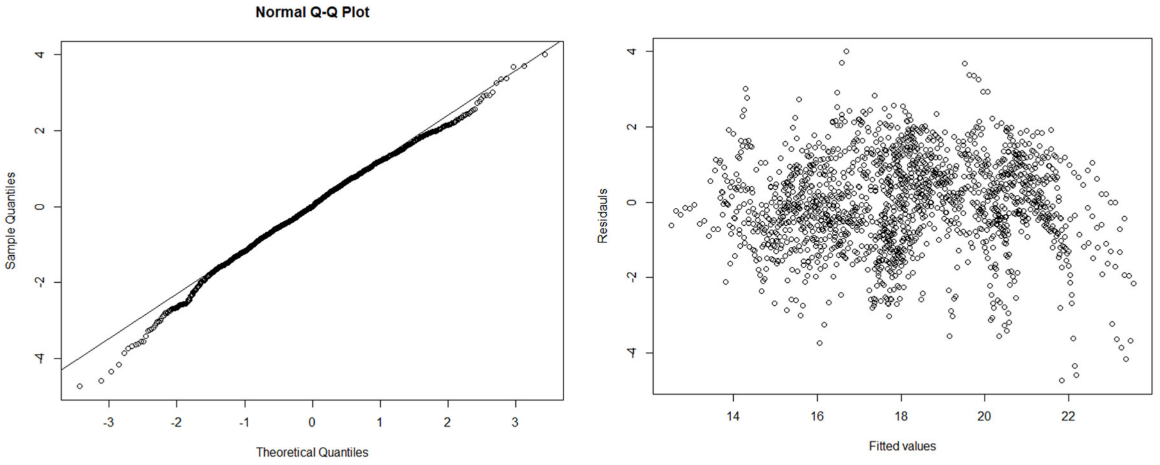

Variable	Sum Sq	Mean Sq	NumDF	DenDF	F value	Pr(>F)
day	658.76	658.76	1	1597.69	435.0588	< 2e-16
condition	5.67	5.67	1	116.18	3.7469	0.05533
day:condition	156.92	156.92	1	1597.69	103.6337	< 2e-16

Day	contrast	estimate	SE	df	t.ratio	p.value
20	F - S	0.438	0.476	81.2	0.92	0.3601
30	F - S	1.159	0.47	76.8	2.467	0.0158
40	F - S	1.879	0.474	79.5	3.965	0.0002
50	F - S	2.6	0.489	89.5	5.321	<.0001
60	F - S	3.32	0.513	108	6.477	<.0001

Supplemental Figure S2: Diagnostic plot for the analysis of bodyweights (Figure 2). A mixed model was used. Left is a normal plot of residuals and right is a fitted value plot. The top table contains the analysis and the bottom table, the linear contrasts . Abbreviations include: Sum Sq (sum of squares), Mean Sq (mean sum of squares), NumDF (degrees of freedom in the numerator), DenDF (degrees of freedom in the denominator), F value (F), Pr(>F) (p value based on f distribution), SE (standard error), df (degrees of freedom), t.ratio (t statistic) and p.value (p value based on t distribution).

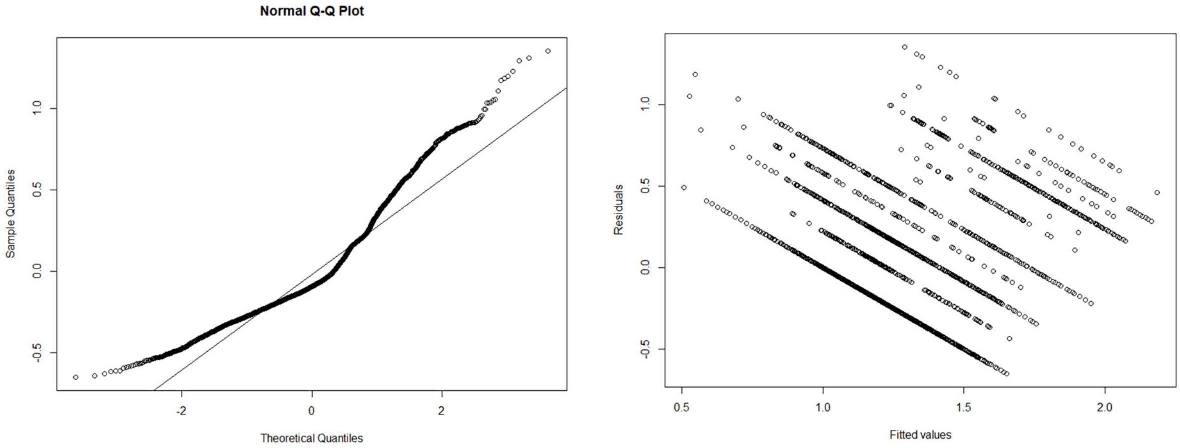

Variable	Sum Sq	Mean Sq	NumDF	DenDF	F value	Pr(>F)
day	87.768	87.768	1	3452.3	826.247	< 2.2e-16
condition	5.151	5.151	1	159.1	48.495	8.24E-11
day:condition	42.139	42.139	1	3452.3	396.697	< 2.2e-16

Day	contrast	estimate	SE	df	z.ratio	p.value
20	F - S	-0.102	0.0284	Inf	-3.585	0.0003
30	F - S	-0.266	0.0297	Inf	-8.944	<.0001
40	F - S	-0.43	0.0331	Inf	-12.981	<.0001
50	F - S	-0.594	0.038	Inf	-15.619	<.0001
60	F - S	-0.758	0.044	Inf	-17.25	<.0001

Supplemental Figure S3: Diagnostic plot for the analysis of clinical scores of infection (Figure 3). A mixed model was used. Left is a normal plot of residuals and right is a fitted value plot. The top table contains the analysis and the bottom table, the linear contrasts . Abbreviations include: Sum Sq (sum of squares), Mean Sq (mean sum of squares), NumDF (degrees of freedom in the numerator), DenDF (degrees of freedom in the denominator), F value (F), Pr(>F) (p value based on f distribution), SE (standard error), df (degrees of freedom), t.ratio (t statistic) and p.value (p value based on t distribution).

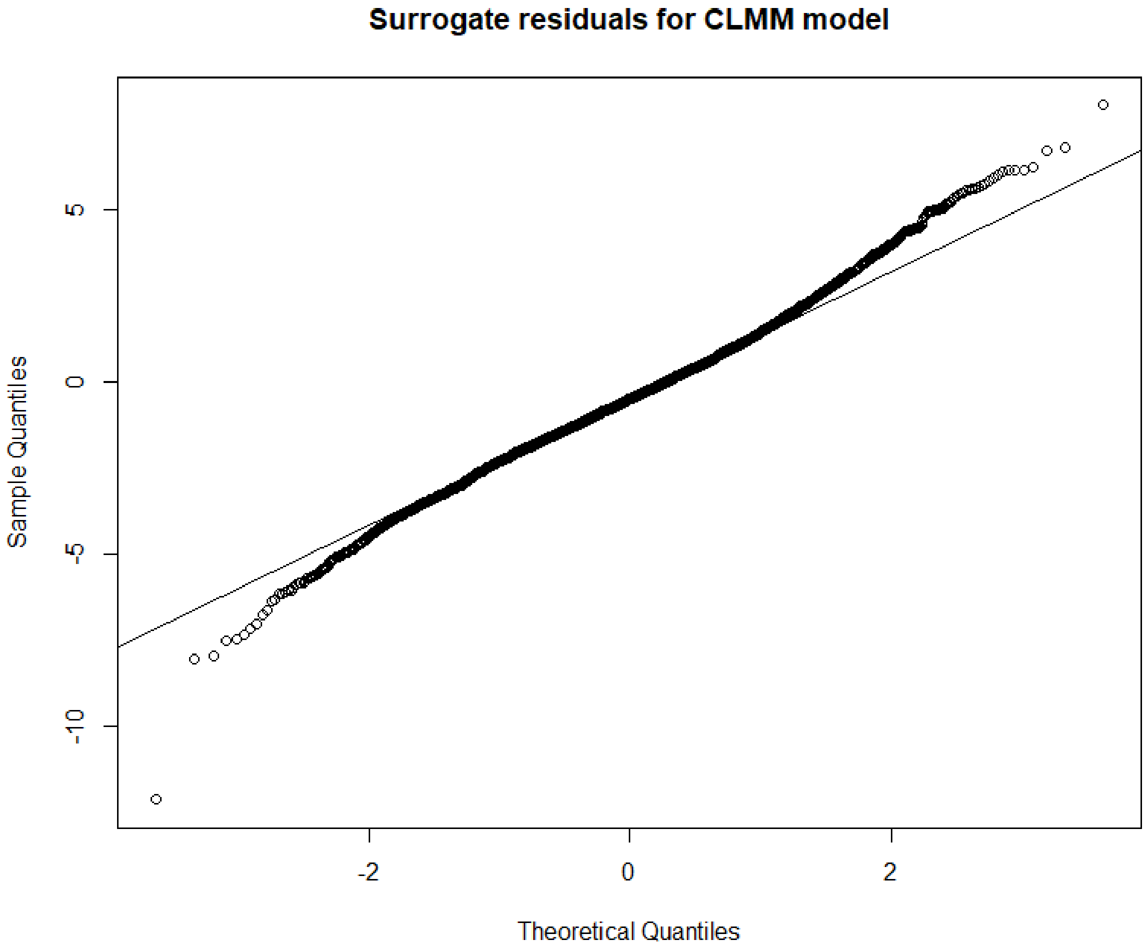

Variable	Df	Chisq	Pr(>Chisq)
day	1	261.414	< 2.2e-16
condition	1	11.007	0.0009075
day:condition	1	372.086	< 2.2e-16

Supplemental Figure S4: Diagnostic plot for the analysis of clinical scores of infection (Figure 4). A cumulative link model was used. Left is a normal plot of residuals and right is a fitted value plot. The top table contains the analysis and the bottom table, the linear contrasts . Abbreviations include: Df (degrees of freedom), Chiq (Chi square value) and Pr(>Chiq) (p value associated with the chi square distribution.

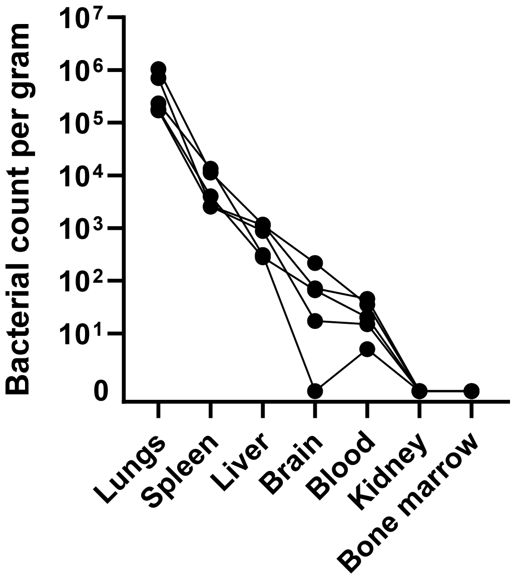

Supplemental Figure S5: The bacterial burden of mice infected with an estimated mean of 142 CFU *of B. pseudomallei bacteria by the inhalational route at 1 day post infection. Bacteria are given as number of bacteria per gram of tissue.*

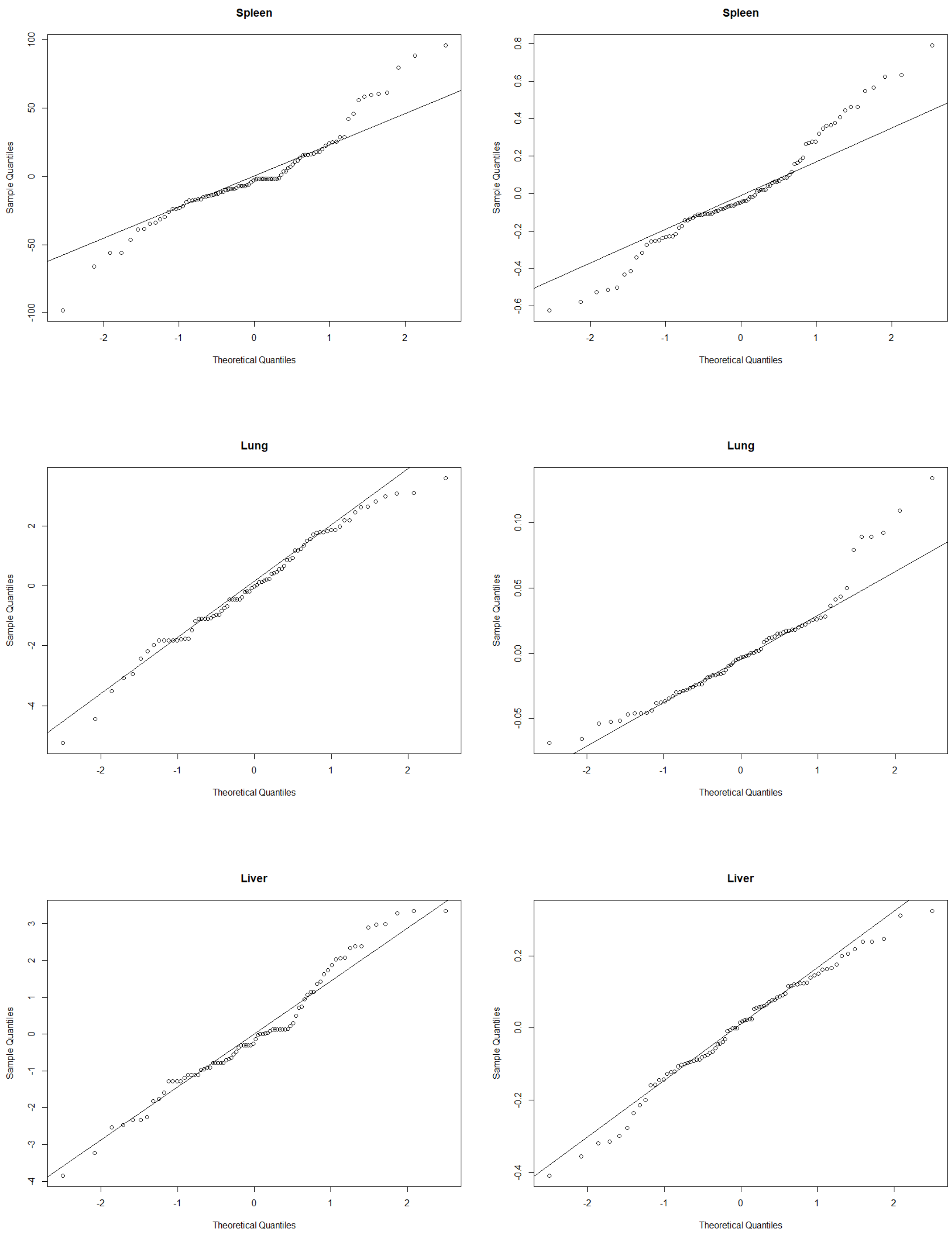
Spleen
Spleen
Lung
Lung
Liver
Liver
Sample Quantiles
Theoretical Quantiles

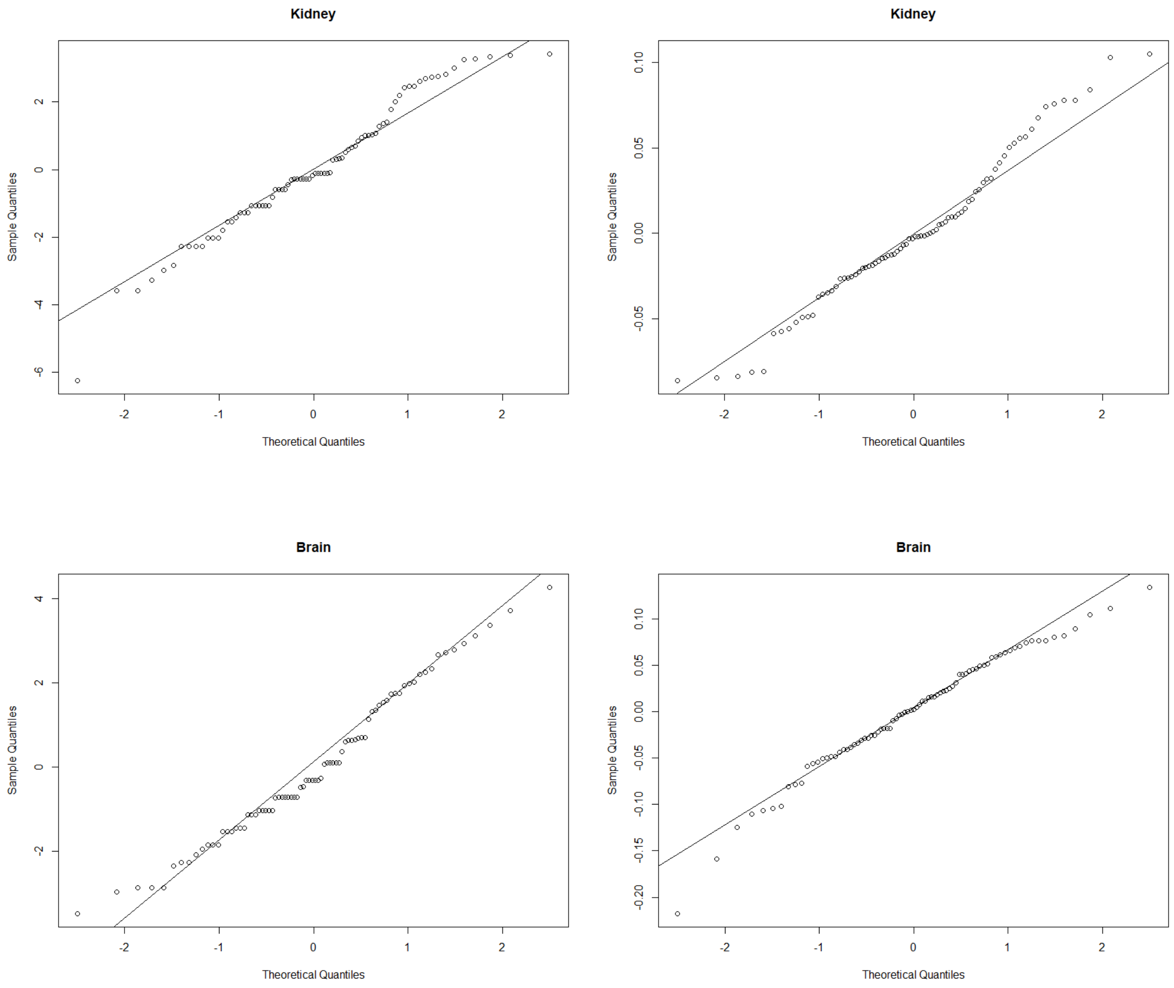

Supplemental Figure S6: Diagnostic plots for the statistical models describe organ weight and bacterial load (Figure 5). Plots on the left are the normal plots of the residuals for the bacterial burden data and the plots on the right are for the organ weight data.

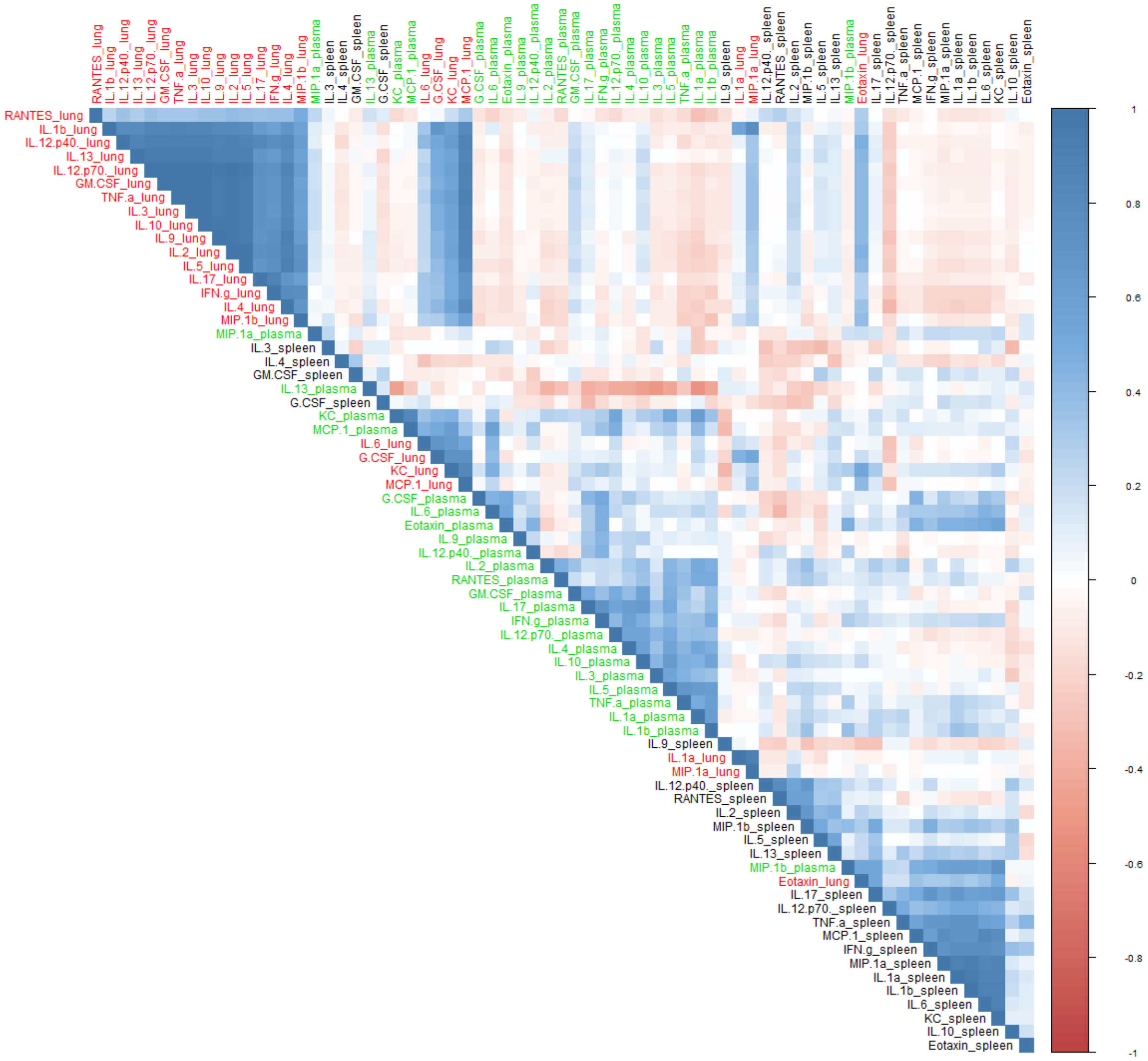

Supplemental Figure S7: A correlation matrix describing the relationship between the concentrations of different cytokines in different organs. Mice were infected with a mean retained dose of 142 CFU of B. pseudomallei by the inhalational route and treated with finafloxacin (37.5 mg/kg) every 8 hours or co-trimoxazole (78 mg/kg) every 12 hours, orally, for 14 days, initiated at 24 hours post-challenge. A group of animals were challenged and treated with the vehicle every 8 hours. The order of reading was derived based on cluster analysis. The colour of each cell is indicative of the Pearson's r.

Supplemental Table S1: Predictive efficacy of the classification algorithms at differentiating animals treated with different antibiotics. Mice were infected with a mean retained dose of $142\ CFU$ of *B. pseudomallei* by the inhalational route and treated with finafloxacin (37.5 mg/kg) every 8 hours or co-trimoxazole (78 mg/kg) every 12 hours, orally, for 14 days, initiated at 24 hours post-challenge. A group of animals were challenged and treated with the vehicle every 8 hours.

Organ	Model	Balanced Acc.	Kappa	Sensitivity	Specificity
Plasma	Rand. Forest	58%	0.17	79%	37%
	Gaus.Proc (Linear)	45%	0.02	44%	46%
	Gaus.Proc (RBF)	62%	0.25	77%	47%
	Gaus.Proc (Poly)	60%	0.19	71%	48%
	SVM (Linear)	53%	0.06	71%	35%
	SVM (RBF)	50%	0	100%	0%
	Logistic GLM	53%	0.17	62%	44%
Lung	Rand. Forest	55%	0.11	73%	37%
	Gaus.Proc (Linear)	45%	0.01	56%	35%
	Gaus.Proc (RBF)	50%	0.02	94%	8%
	Gaus.Proc (Poly)	58%	0.11	67%	43%
	SVM (Linear)	55%	0.09	61%	48%
	SVM (RBF)	50%	0	100%	0%
	Logistic GLM	58%	0.16	66%	50%
Spleen	Rand. Forest	57%	0.14	76%	37%
	Gaus.Proc (Linear)	55%	0.1	61%	49%

	Gaus.Proc (RBF)	54%	0.1	88%	20%
	Gaus.Proc (Poly)	60%	0.12	72%	47%
	SVM (Linear)	49%	0.03	63%	33%
	SVM (RBF)	50%	0	100%	0%
	Logistic GLM	61%	0.22	74%	49%

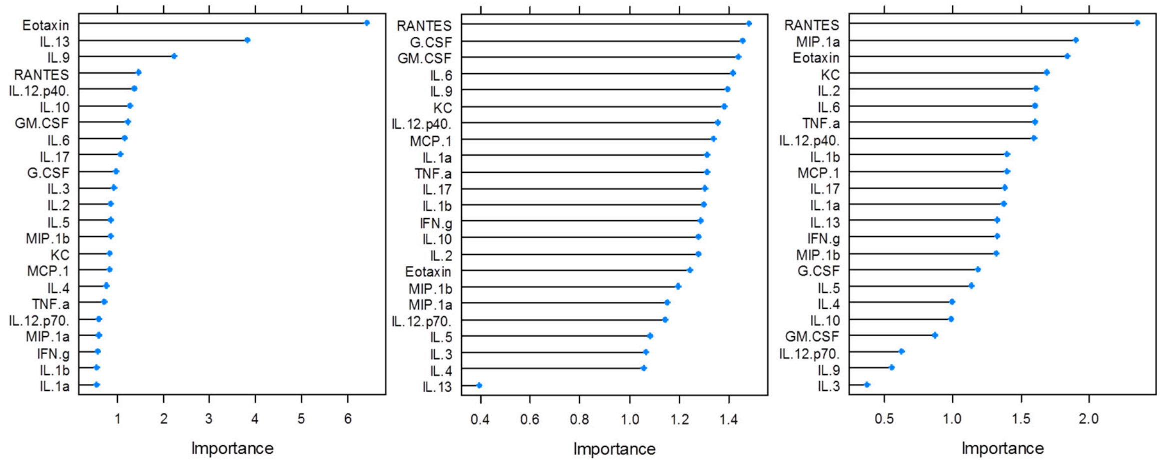

Supplemental Figure S8: The relative importance of a panel of cytokines estimated by a Random Forest classifier. Luminex assays were performed on samples harvested throughout the experiment and the levels of a panel of cytokines determined. The random forest algorithm was selected to rank input dimensions (cytokines) by their variable importance to determine which cytokines reliably differentiated the two antibiotics treatment regimens.

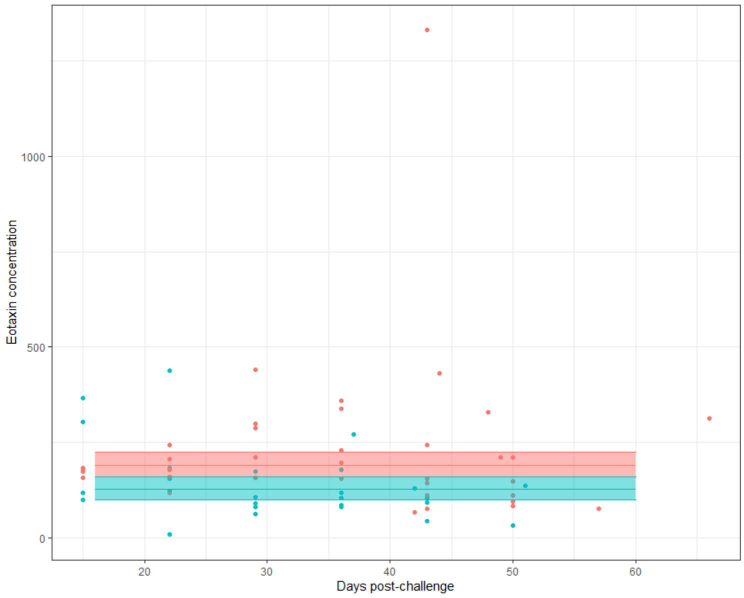

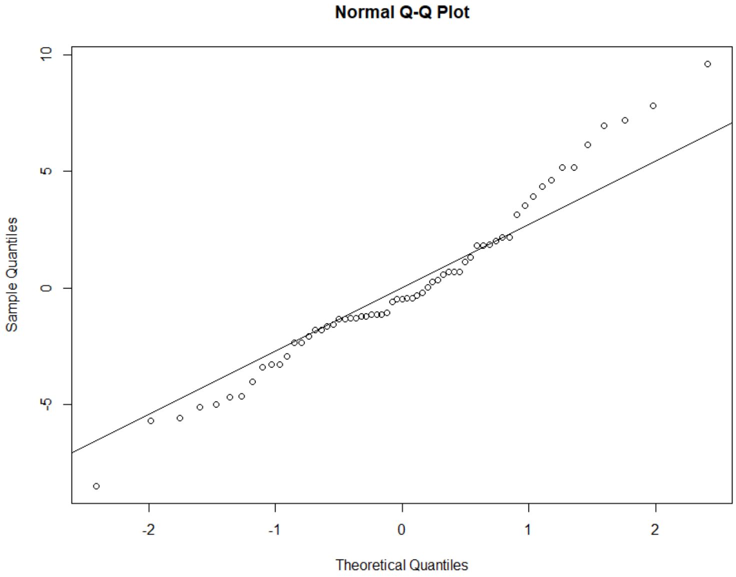

Supplemental Figure S9: The Eotaxin levels recorded following antibiotic treatment in the lung. Mice were infected with an estimated mean retained dose of 142 *CFU of B. pseudomallei via the inhalational route and treated from 24 hours post-challenge with finafloxacin (37.5 mg/kg) (red) every 8 hours or co-trimoxazole (78 mg/kg) (blue) every 12 hours delivered by the oral route for 14 days. A group of animals were challenged and treated with the vehicle every 8 hours. The shaded area represents the 95% prediction interval, the dots represent the original data points. Red - finafloxacin, blue - co-trimoxazole, grey - indicates overlapping data from both antibiotics. The lower plot is a diagnostic plot for statistical model.*

Supplemental Table S2: Summary of analysis of the cell count data. Mice were infected with a mean retained dose of 142 CFU of B. pseudomallei via the inhalational route and treated with finafloxacin (37.5 mg/kg) every 8 hours or co-trimoxazole (78 mg/kg) every 12 hours, orally, for 14 days, initiated at 24 hours post-challenge. A group of animals were challenged and treated with the vehicle every 8 hours. A Bonferroni's correction was applied to account for the 13 analyses.

**Measurement**	**Tissue**	**Effect of time**	**Effect of treatment**	**Rate of change**
Neutrophils	Lung	P=0.169	P>0.999	P=0.208
Granulocytes	Lung	P>0.999	P>0.999	P>0.999
Lymphocytes	Lung	P>0.999	P>0.999	P>0.999
Granulocytes CD64+	Lung	P=0.013 *	P>0.999	P>0.999
Granulocytes MHC Class II	Lung	P=0.299	P>0.999	P>0.999
Neutrophils	Spleen	P<0.013 *	P>0.999	P>0.999
Granulocytes	Spleen	P=0.065	P<0.013 *	P<0.013 *
Granulocytes CD80+	Spleen	P>0.999	P=0.884	P>0.999
Neutrophils	Plasma	P<0.013 *	P>0.999	P>0.999
Granulocytes	Plasma	P<0.013 *	P>0.999	P>0.999
Lymphocytes	Plasma	P<0.013 *	P>0.999	P>0.999
Granulocytes CD64+	Plasma	P=0.065	P>0.999	P>0.999
Granulocytes MHC Class II	Plasma	P=0.026 *	P>0.999	P>0.999

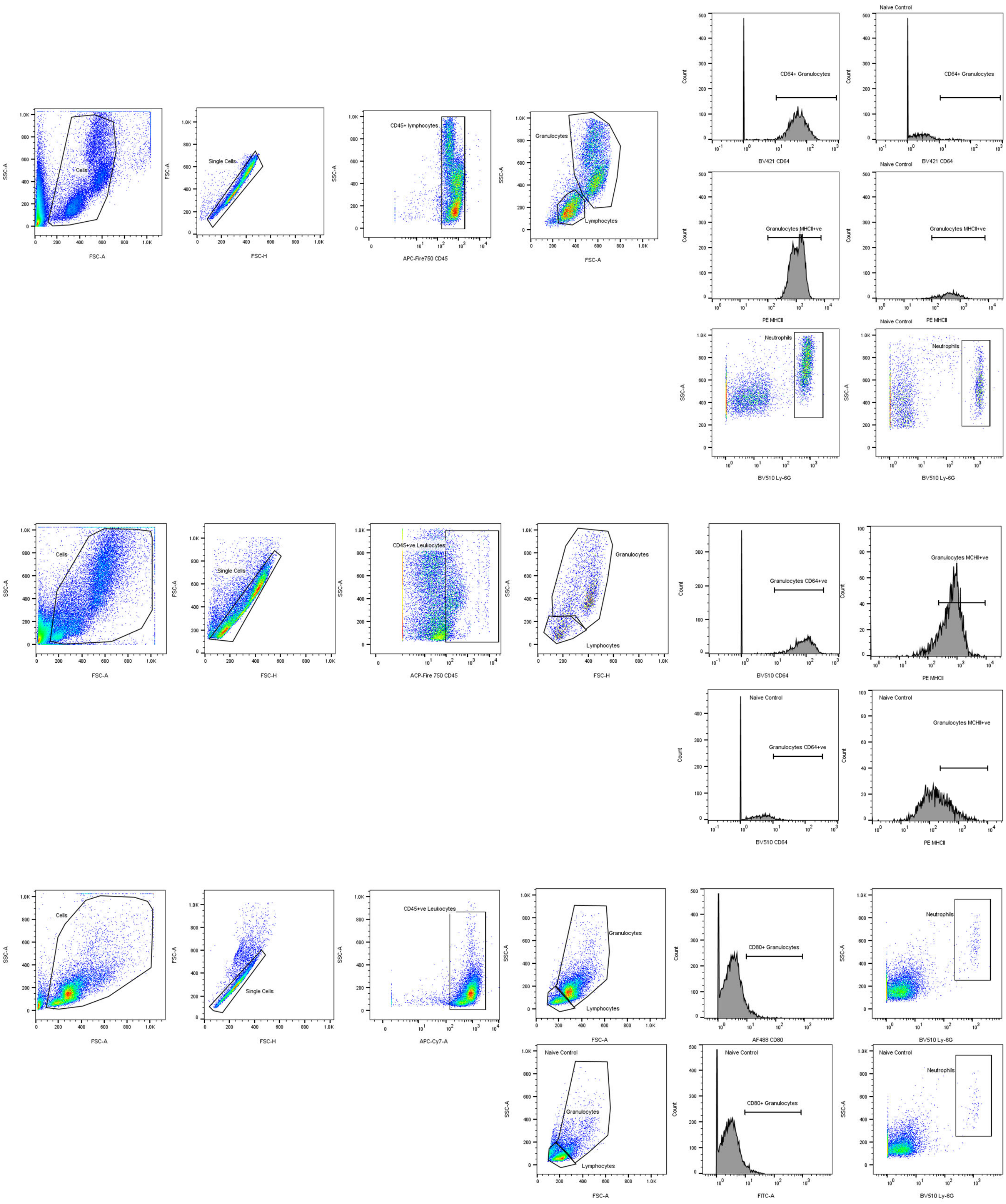

Supplemental Figure S10: The gating strategy used in the identification and numeration of leukocytes cells in this study. Top shows the strategy for the blood, middle for the lung, bottom for the spleen. The gates are shown as sub gates to the one shown to the left.
